# HIV Pre-exposure Prophylaxis Implant Stated Preferences and Priorities: Results of a Discrete Choice Experiment Among Women and Adolescent Girls in Gauteng Province, South Africa

**DOI:** 10.1007/s10461-022-03658-w

**Published:** 2022-03-31

**Authors:** Kristen M. Little, Lola Flomen, Homaira Hanif, Sharon M. Anderson, Andrea R. Thurman, Meredith R. Clark, Gustavo F. Doncel

**Affiliations:** 1 HIV/TB Department, Population Services International (PSI), Washington, DC USA; 2 Strategy & Insights Department, PSI, 1120 19th Street NW, Suite 600, Washington, DC 20036 USA; 3 CONRAD, Eastern Virginia Medical School, Norfolk, VA USA

**Keywords:** Discrete choice experiment, Pre-exposure prophylaxis, HIV, South Africa, Experimento de elección discreta, Profilaxis previa a la exposición, VIH, Sudáfrica

## Abstract

For adolescent girls (AG) and young women (YW), adherence barriers may limit the effectiveness of daily oral HIV pre-exposure prophylaxis (PrEP). Due to its low-burden and long-lasting product attributes, PrEP implants could remove some of the critical adherence barriers of oral PrEP products for individuals at risk of HIV. To explore stated preferences for a long-acting PrEP implant, we conducted a quantitative survey and discrete choice experiment with AG (ages 15–17), YW (18–34), and female sex workers (FSW; ≥ 18) in Gauteng Province, South Africa. We completed 600 quantitative surveys across the three subgroups of women. Respondents stated preference for an implant that provided longer HIV protection (24 months versus 6 months) and required a single insertion. They stated that they preferred a biodegradable implant that could be removed within 1 month of insertion. Respondents had no preference for a particular insertion location. Overall, 78% of respondents said they would be likely (33%) or very likely (45%) to use a PrEP implant were one available, with the majority (82%) stating preference for a product that would provide dual protection against HIV and unintended pregnancies. To reduce their risk of HIV, AG, YW, and FSW in our survey reported a strong willingness to use long-acting, highly-effective, dissolvable PrEP implants.

**Supplementary Information:**

The online version contains supplementary material available at 10.1007/s10461-022-03658-w.

## Resumen

Las niñas adolescentes (NA) y mujeres jóvenes (MJ), pueden enfrentar barreras de adherencia que limitan la eficacia de la profilaxis oral previa a la exposición al VIH (PrEP). Ya que el implante de PrEP es un producto que requiere de poca intervención de la usuaria y es de larga duración, podría eliminar algunas de las barreras de adherencia más importantes en el uso de los productos orales de PrEP para aquellas personas en riesgo de infección de VIH. Para explorar las preferencias declaradas en cuanto al implante de PrEP de acción prolongada, llevamos a cabo una encuesta cuantitativa y un experimento de elección discreta (DCE) con NA (de 15 a 17 años), MJ (de 18 a 34 años) y mujeres trabajadoras del sexo (MTS; ≥ 18 años) en la provincia de Gauteng, Sudáfrica. Administramos 600 encuestas cuantitativas en los tres subgrupos de mujeres. Los resultados indican la preferencia por un implante que proporciona una protección contra el VIH más prolongada (24 meses a comparación con 6 meses) y que requiere de una única inserción. Las participantes afirmaron que prefieren un implante biodegradable que puede retirarse un mes después de su inserción. Las participantes no tenían preferencia por un sitio específico de inserción. En general, el 78% de las participantes indicaron que probablemente (33%) o muy probablemente (45%) utilizarían un implante de PrEP si estuviera disponible, y la mayoría (82%) manifestó su preferencia por un producto que proporcionaba una doble protección contra el VIH y el embarazo no deseado. Para reducir el riesgo de contraer el VIH, las NA, MJ y MTS participantes se mostraron muy dispuestas a utilizar implantes de PrEP de larga duración, altamente eficaces y disolubles.

## Background

The HIV epidemic in South Africa disproportionally impacts young women (YW) under the age of 30 [1], who have an HIV prevalence of 17%, approximately 2.5 times higher than that of men in the same age group [2, 3]. Young women are at particularly high risk of contracting HIV, and have the highest HIV incidence amongst all age segments [4, 5]. Among YW, female sex workers (FSW) and those engaged in transactional sex are at particularly high risk for HIV with a prevalence ranging from 48 to 72% [6, 7].

While biomedical HIV prevention options such as oral PrEP are increasingly available in South Africa, women in particular, face barriers to oral PrEP access, uptake, and continuation. Barriers, including frequent visits to health clinics and challenges in taking medication on a daily basis [8–11], can have a negative impact on adherence of oral PrEP, diminishing the effectiveness against HIV acquisition. In a recently published study, an injectable antiretroviral was more effective (HR 0.34) than oral PrEP in preventing HIV seroconversions in cisgender men and transgender women [12]. Similar results were obtained in cisgender women [13]. A likely explanation for this difference is poorer or inconsistent adherence to the oral PrEP regimen. Furthermore, a number of studies have found that oral PrEP is comparatively less effective among women than among men, especially at lower levels of adherence [14–19]. South Africa has a strong enabling environment for increasing young women’s access to biomedical HIV prevention products [20–22], including the National Strategic Plan for HIV/TB, which contains PrEP guidance and provision for at-risk youth populations [23–25]. Given the barriers to oral PrEP uptake and adherence, however, additional biomedical prevention options are needed to reduce the burden of HIV among women and girls in this context.

Along with long-acting injectables and vaginal rings, long-acting implants are being developed [26]. A biodegradable implantable PrEP (PrEP implant) has the potential to give those at high risk for HIV additional choices for biomedical HIV prevention, and to reduce HIV incidence and clinic visits in high-burden settings [27, 28]. In-depth interviews with healthcare providers in South Africa demonstrate that with adequate training, providers could offer at-risk clients the PrEP implant as an alternative to daily oral PrEP [29]. While promising, initial testing of the PrEP implant, including target end-user stated preferences for implant features, has been limited [30, 31].

**Fig. 1:**
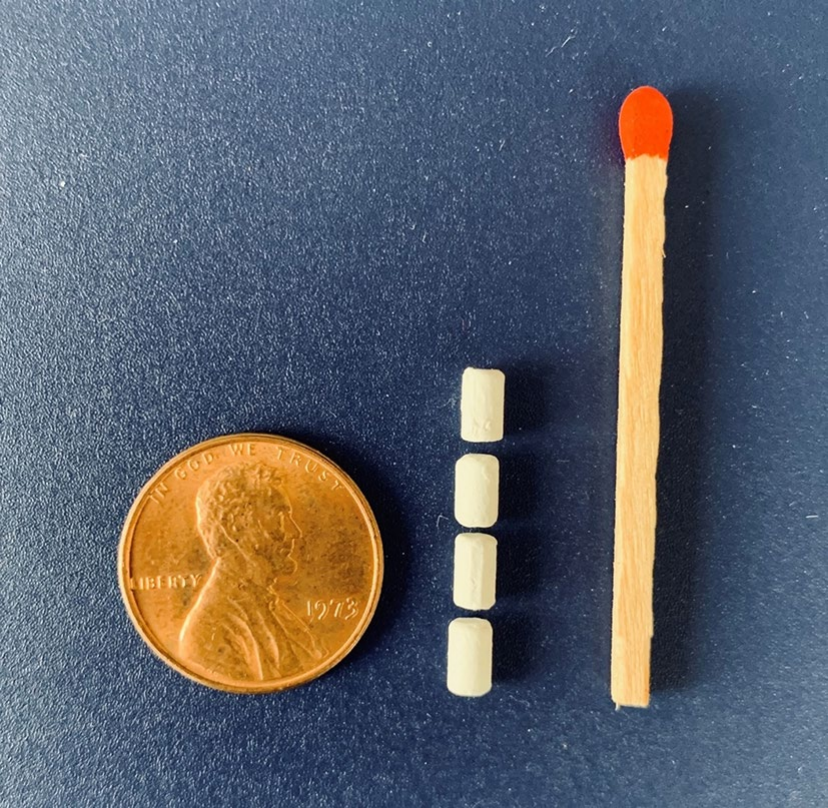
ARV-releasing pellet prototype, under development by CONRAD

To better understand end-user preferences for a potential PrEP implant, we conducted a quantitative survey and discrete choice experiment (DCE) to evaluate perceptions and product preferences for the PrEP implant amongst adolescent girls (AG), YW, and FSW in Gauteng Province, South Africa. This study is the first to focus on gaining insights into the acceptability and stated preferred product attributes of an innovative antiretroviral -releasing pellet implant design (Fig. 1) among different population segments of young women at risk of acquiring HIV in South Africa.

## Methods

### Study Setting and Population

The survey was conducted in rural and urban settings in Gauteng Province, South Africa. The sites in Gauteng were purposively selected to include geographies with ongoing oral PrEP interventions, and where the target populations were relatively more familiar with oral PrEP [32]. For a diversity of respondents, the primary study site was the Soshanguve township near Pretoria—an area with varied levels of education, income, and access to healthcare [31]. Respondents were eligible for the study if they were: AG ages 15–17, YW aged 18–30, or FSW over the age of 18, were sexually active, currently resided in the study area, and were able/willing to provide informed consent.

Based on accepted approaches to DCE sample sizes, we estimated we would need approximately 200 respondents per population segment for this study [33, 34]. Our sample size of 600 participants was divided across the three study groups (AG, YW, and FSW) in both rural and urban settings. AGYW were randomly sampled from households during a multi-stage household survey. FSW were selected through a respondent driven sampling with seeds identified in bars and shebeens (unlicensed location selling alcohol). More information on the sampling approach is available in the Supplemental Materials.

### Quantitative Survey and Discrete Choice Experiment

The quantitative survey collected data on socio-demographic characteristics, awareness and use of analogous products including the contraceptive implant and daily oral PrEP, HIV risk perception, and current HIV prevention approaches (including abstinence, partner reduction, condom use, partner testing, and/or oral PrEP). Willingness to pay for oral PrEP and the PrEP implant was also included, using a contingent evaluation approach.

**Fig. 2:**
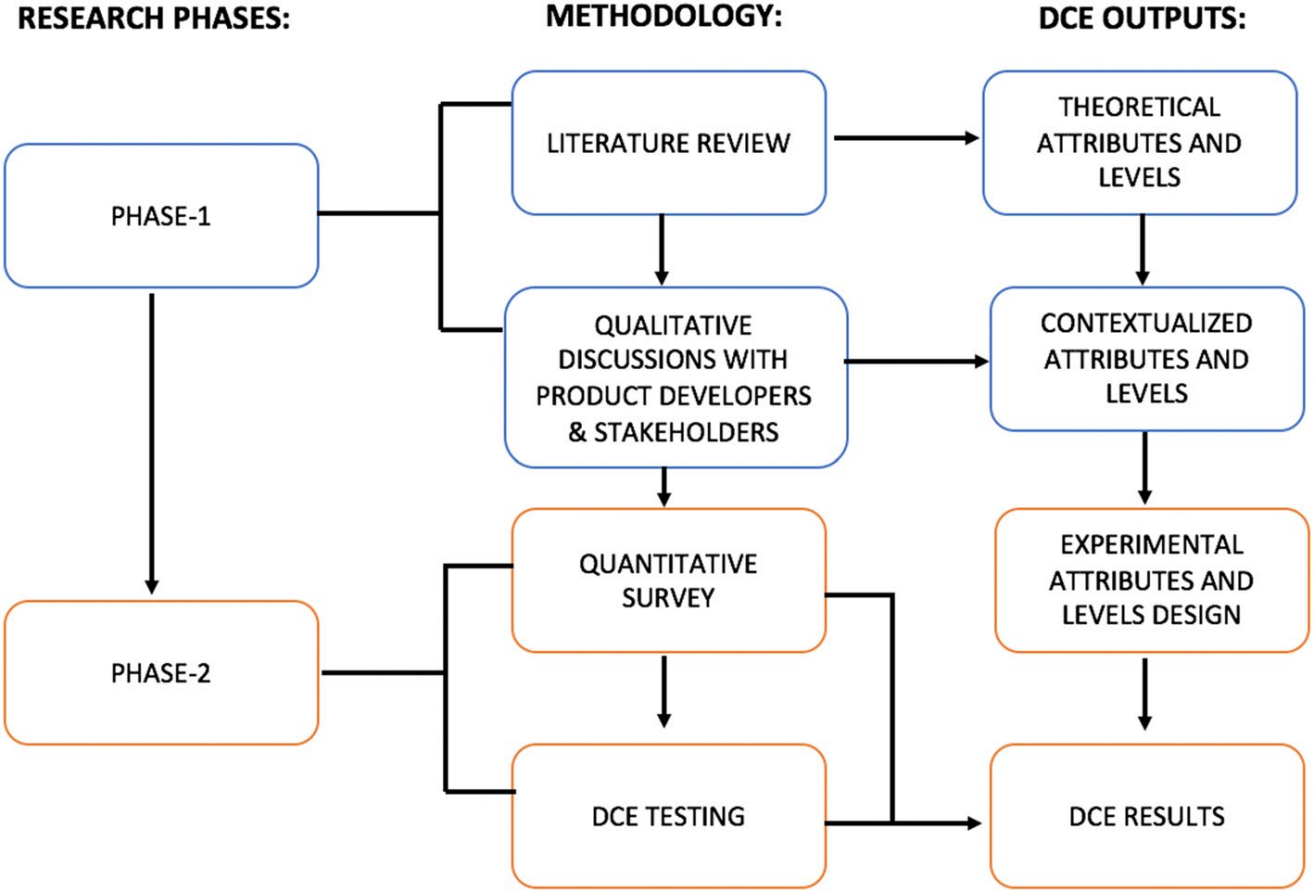
Process of determining choice task attributes and levels. *Note* Schematic of Research Phases 1 and 2 adapted from “Developing attributes and attribute-levels for a discrete choice experiment on micro health insurance in rural Malawi” by Abiiro, G., Leppert, G., Mbera, G. et al. 2014, BMC, copyright by BMC

**Fig. 3:**
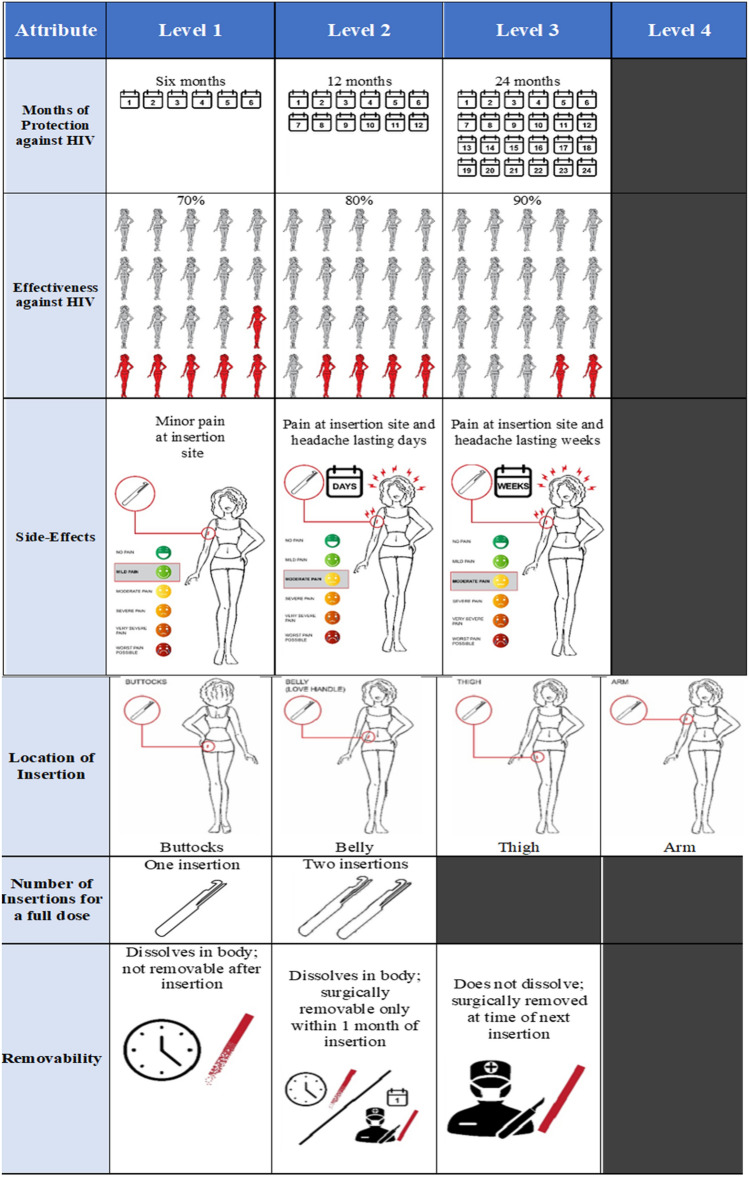
Discrete choice experiment attributes and levels

The DCE design was informed by a literature review, input from the product developer, stakeholder engagement, and qualitative formative research with target end-users (Fig. 2). Ngene software was used to develop the experiment design using a D-efficient design approach for analysis of main effects while minimizing attribute level balance, overlap and dependence. The resulting design contained six attributes, with two to four possible levels each (see Fig. 3). To limit cognitive fatigue, each participant was randomized to one block of 8 choice sets during the survey.

### Study Design and Statistical Analysis

Participants completed the quantitative survey and DCE with a trained interviewer using a tablet. Data were collected and stored in SurveyCTO. During the DCE, participants were presented choice tasks with illustrative graphics. For each choice task, participants selected their preference between the two unlabeled product options and then indicated if they would rather use the option selected or their current HIV prevention approach.

Quantitative survey data were analyzed descriptively using means and frequencies and appropriate tests of association (e.g. t tests and chi-square tests). The probability of living in poverty was estimated using the Probability Poverty Index [35]. Stated preference data were analyzed using fixed effects logistic regression in Stata 15.0 (College Station, TX). Stratification analyses were conducted to determine whether differences in stated preferences existed between end-user groups. Differences in stated preference estimates by strata were evaluated using Chow and Wald tests [36].

### Ethics

The University of the Witwatersrand Human Research Ethics Committee (Medical) and the Population Services International Research Ethics Board (PSI REB) granted the necessary ethical and regulatory approval and each respondent provided written informed consent prior to their participation (FSW provided verbal consent to participate).

## Results

Between March 7–23, 2020, we recruited 600 participants, including 201 AG, 200 YW, and 199 FSW. Half of the study population lived in urban settings and the remaining half resided in rural, peri urban and urban areas of Gauteng province (Table 1). More than half (52.4%) of the sample reported ever having been pregnant, though this varied from 7% among AG to 70% among YW and 81.4% among FSW (Pearson’s $\chi^{2}$ p < 0.001). YW had significantly higher levels of education than FSW or AG, with 68.8% having completed Metric education or above (beyond secondary education), versus 13.9% of AG and 36.8% of FSW (Pearson’s $\chi^{2}$ p < 0.001).

**Table 1:** Sociodemographic characteristics by study population

Characteristic	Total N = 600, (%) ​	Adolescent girls N = 201, (%) ​	Young women​ N = 200, (%)	Female sex workers N = 199​, (%)	P value
Age ​ (Mean, SD)	24.2 (8.0)	16.1 (1.0)	25.5 (3.6)	31.2 (7.7)	< 0.001
Single/not cohabitating	523 (88.6)	201 (100.0)	156 (78.4)	166 (87.4)	< 0.001
Ever been pregnant	312 (52.4)	14 (7.0)	140 (70.0)	158 (81.4)	< 0.001
*Education*					
Primary	201 (33.9)	53 (26.4)	2 (2.6)	23 (11.9)	< 0.001
Secondary	199 (33.6)	120 (59.7)	60 (30.2)	99 (51.3)	
Metric or above	193 (32.6)	28 (13.9)	137 (68.8)	71 (36.8)	
Currently employed	249 (41.9)	2 (1.0)	65 (33.3)	182 (91.5)	< 0.001
Average monthly income	8183 (14,651)	4650 (4455)	5918 (6246)	9275 (17,185)	0.252
Probability poverty index	55.2 (15.2)	56.4 (16.5)	54.0 (16.9)	55.2 (11.5)	0.430

### Quantitative Survey

#### Experience with Analogous Products

While most respondents (68%) had heard of contraceptive implants, only 18% of the study sample reported having ever used a contraceptive implant. Experience with the implant was higher among FSW (29%) than YW (20%) or AG (5%, Pearson’s $\chi^{2}$ p < 0.001). Implant experience did not vary significantly between urban (19%) and rural/peri urban (17%) settings (Pearson’s $\chi^{2}$ p = 0.387). Awareness of oral PrEP was low amongst respondents (33%). This was especially true for AG (18%) and YW (32%), though awareness was higher among FSW (51%, Pearson’s $\chi^{2}$ p < 0.001). The respondents who were aware of oral PrEP had a higher average perceived risk of acquiring HIV (5.9 vs. 3.8, t test p < 0.001). Even amongst those aware of oral PrEP, use of the product remained low, at just 20%. This ranged from 24% among PrEP-aware FSW to just 20% among AG and 14% among YW (Pearson’s $\chi^{2}$ p = 0.330).

#### Service Delivery Stated Preferences

While 66% of respondents said they would be “likely” or “very likely” to take oral PrEP in the future, only 34% of respondents had knowledge of where to access oral PrEP. As with oral PrEP awareness, knowledge of where to access oral PrEP was higher amongst FSW (47%) than YW (34%) or AG (21%, Pearson's $\chi^{2}$ p < 0.001). Across the respondent sub-groups, hospitals and clinics were the stated preferred channels for accessing oral PrEP.

After hearing about the PrEP implant, the majority of respondents said they would be “likely” (33%) or “very likely” (45%) to use it were it available. The likelihood of using the PrEP implant was particularly high for both FSW (86%) and YW (81%) compared to AG (67%, Pearson’s $\chi^{2}$ p = 0.002). Most respondents (55%) desired to receive the PrEP implant from a family planning (FP) provider in comparison to an HIV care provider, a preference that did not differ significantly by sub-group (Pearson’s $\chi^{2}$ p = 0.411). The majority of respondents (82%) preferred a product that would provide dual protection against HIV and unintended pregnancies. Data show that respondents on balance would prefer a product that led to fewer and more short-lived side effects. Adjusting for other product features, respondents had 2 (95% CI 1.72–2.22) times the odds of choosing a product that caused temporary pain at the injection site rather than one that caused pain at the injection site, muscle pain, and headaches lasting for weeks.

#### PrEP Implant Product Stated Preferences

Across the sub-groups, respondents stated a preference for a long-acting, dissolvable implant product. Importantly, 72% of respondents said they would prefer a product that dissolved and didn’t require surgical removal. Respondents also preferred longer-acting PrEP implants over shorter-acting PrEP injectable products: 83% of respondents preferred 12-month implants to 3-month injectables (Table 2).

**Table 2:** Attribute preferences from DCE

Attribute	Level	Utility ratio	95% CI
Duration of protection	6 months	Ref	Ref
	12 months	1.07	(0.94–1.22)
	24 months	**1.42**	**(1.25–1.61)**
Effectiveness	70%	Ref	Ref
	80%	**4.35**	**(3.84–4.94)**
	90%	**12.88**	**(11.25–14.75)**
Side effects	Pain at injection site	**1.95**	**(1.72–2.22)**
	Pain at injection site & headache lasting days	**1.22**	**(1.06–1.40)**
	Pain at injection site & headache lasting for weeks	Ref	Ref
Location of insertion	Buttocks/thigh	Ref	Ref
	Belly/love handle	1.06	(0.91–1.23)
	Arm	1.05	(0.92–1.20)
Number of insertions	One	**1.44**	**(1.30–1.58)**
	Two	Ref	Ref
Dissolvability	Not dissolvable, surgical removal	Ref	Ref
	Dissolvable, not removable	0.93	(0.81–1.06)
	Dissolvable, removable within 1 month	**1.20**	**(1.05–1.36)**

For dissolvable, removable within 1 month = *p-value < 0.05 For number of insertions, one = ***p-value < 0.001 For side effects, pain at injection site & headache lasting days = **p-value < 0.01 For side effects, pain at injection site = ***p-value < 0.001 For effectiveness at 90% = ***p-value < 0.001 For effectiveness at 80% = ***p-value < 0.001 For duration, 24 months = ***p-value < 0.001

### Discrete Choice Experiment

**Fig. 4:**
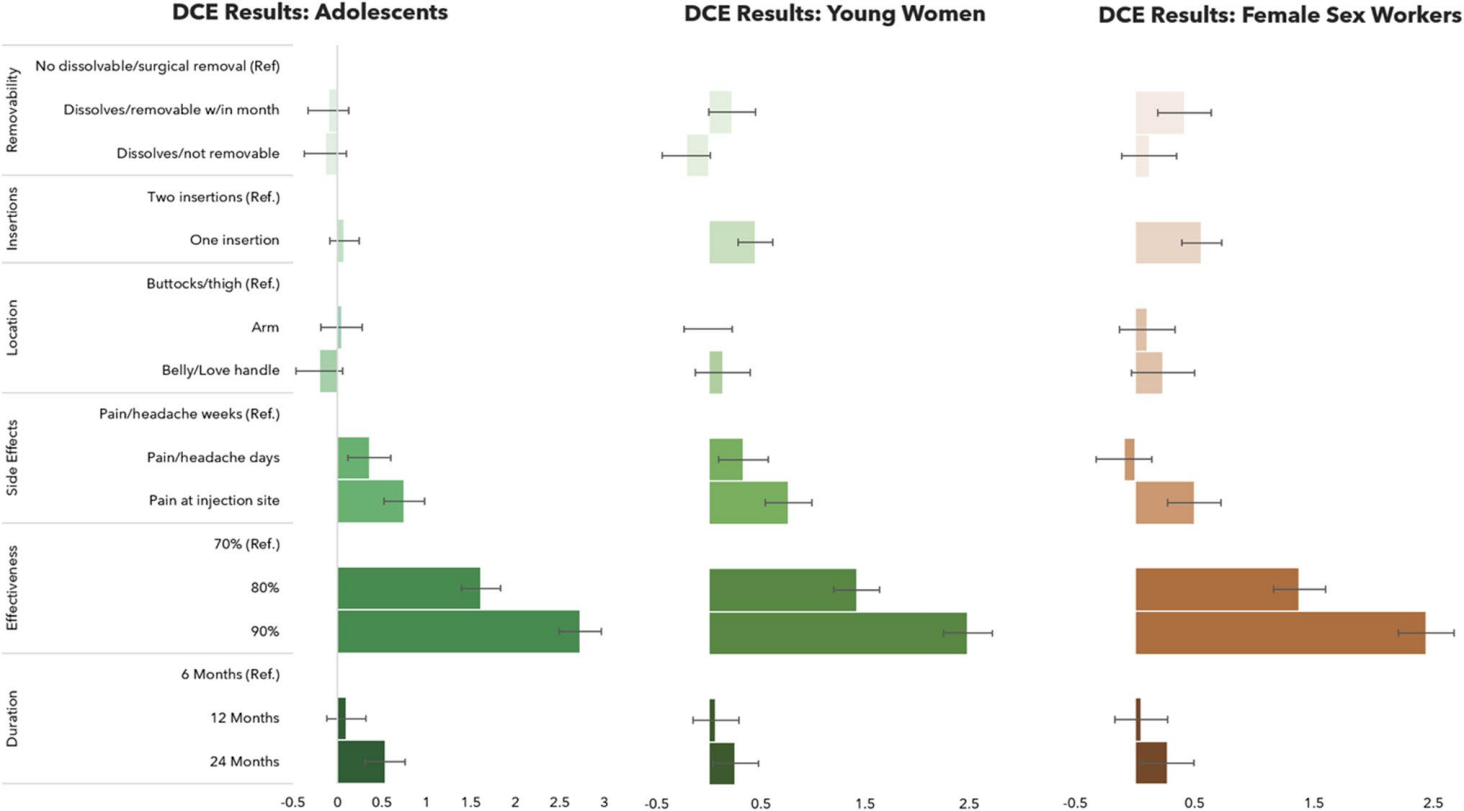
DCE results by population segment

Among all respondents, product effectiveness, side effects, duration of protection, number of insertions, and product removability were all significantly associated with PrEP implant product choice (Fig. 4). Only location of insertion was not significantly associated with product preference after adjusting for other attributes. Respondents were more likely to select a product with higher prevention efficacy (Fig. 4). Compared to a product providing 6 months of HIV protection, respondents had 1.4 (95% CI 1.2–1.6) times the odds of selecting a product that provided 24 months of protection and 1.1 (95% CI 0.9–1.2) times the odds of selecting a 12-month protection product. Respondents also stated preference for products that required one insertion vs. one that required two separate insertion sites [Utility Ratio (UR) 1.5, 95% CI 1.3–1.6]. While dissolvability was preferred over a product that needed to be surgically removed, this was only significant for dissolvable products that had a window of removability (UR 1.2, 95% CI 1.05–1.4) that lasted up to 1-month post insertion.

Stated preferences were generally similar across the three study sub-groups, especially between YW and FSW where no significant differences in attribute preferences were observed (Fig. 4). Some significant differences emerged when comparing preferences between AG and YW, where AG showed stronger stated preferences for a product with a 90% effectiveness relative to one with 70% effectiveness against HIV (UR 15.3 vs. 11.9, Wald $\chi^{2}$ p = 0.048) and a 24-month product vs. a 6-month product (UR 1.7 vs. 1.3, Wald $\chi^{2}$ p = 0.022). AG were less driven by preferences for one insertion versus two insertions relative to their older YW counterparts (UR 1.1 vs. 1.6, Wald $\chi^{2}$ p < 0.001).

While preferences across the study population groups were generally consistent, significant differences in preferences were observed between urban and rural/peri-urban respondents. AG, YW and FWS in urban areas had stronger stated preferences for implants that lasted for 24 months versus the 12-month option than respondents in rural and peri-urban settings (UR 0.49 vs. 0.22, Wald $\chi^{2}$ p = 0.0151). Respondents in urban settings also had stronger preferences for one insertion versus two insertions to respondents in rural and peri-urban settings (UR 0.43 vs. 0.29, Wald $\chi^{2}$ p = 0.0195). In contrast, respondents in rural and peri-urban settings had stronger stated preferences for implants that are dissolvable but removable within 1 month versus their urban counterparts. (UR 0.27 vs. 0.08, Wald $\chi^{2}$ p = 0.0094).

## Discussion

This research was intended to primarily inform product development, while providing additional market segmentation information. Findings from this study, as well as an expanded DCE study currently in progress, will be integrated into the target product profile to guide final product development. While there is a growing body of research on oral PrEP DCEs conducted in South Africa [9, 36–39], this is among the first studies to evaluate priorities and preferences of implantable PrEP across different female population segments [8, 29, 37]. While AG in South Africa are frequently targeted with HIV prevention social and behavior change campaigns (SBC) [40–42], it is not common for adolescents to be the target population for DCEs [35, 43]. To better explore barriers to youth uptake of HIV prevention products, a number of studies have called for the increased incorporation of adolescent populations into DCEs [20, 31, 44, 45]. This study highlighted the importance of understanding adolescents’ stated preferences as end-users of HIV biomedical prevention products. Despite the availability of oral PrEP in South Africa, consistent with other studies determining oral PrEP awareness, AG and YW were significantly less aware of the product than FSW respondents [46–48]. While South Africa has policies to improve AG and YW access to oral PrEP, the product was not initially marketed to those priority groups [24]. Oral PrEP in South Africa was originally targeted primarily at FSW, and our results are consistent with other studies that have found a higher awareness of oral PrEP among FSW compared to other segments [49].

Notwithstanding the low awareness of oral PrEP, respondents expressed interest and a high likelihood of using a PrEP implant once the product concept was explained to them, especially among the older age groups. YW have demonstrated a willingness to use daily oral PrEP across sub-Saharan Africa, and more recent research has also shown the acceptability of other biomedical prevention options including monthly PrEP vaginal rings and long-acting injectable products [50, 51]. While stated acceptability of oral PrEP products is high among YW, studies in South Africa have demonstrated that this group is more likely to adopt HIV prevention products that blend into their lifestyles [51]. While YW may be willing to use oral PrEP, the daily dosage requirement is often viewed as inconvenient and not-discreet, which can limit uptake and continuation [10]. Similarly, due to frequent visits to providers and concerns about male partner reactions, the monthly PrEP vaginal ring has also been perceived by some women as inconvenient [52]. Consistent with other studies on biomedical HIV prevention products in South Africa, this study demonstrated that young women prioritize products that are: (i) highly effective in preventing HIV, (ii), long-lasting and (iii) discreet [51]. The findings show that the PrEP implant has the potential to address the barriers to oral and vaginal ring PrEP products due to its assumed high effectiveness, convenience, long-lasting protection, and discreet nature.

As in other studies [29, 38], women and girls in our survey significantly valued high product effectiveness and long duration of protection. In this study population, these two attributes have not been associated with oral PrEP. In our study, however, other modifiable product characteristics emerged as important to target end-users. Dissolvability, relative to a product that required surgical removal (analogous to the currently available contraceptive implants) appealed to end-users. This is important because all contraceptive implants and most PrEP implants in development are non-biodegradable and require removal [29]. However, this was only true for degradable products that additionally had a short initial window of removability. This finding was further supported by the results of qualitative formative research [53], which found that end-users had concerns about a fully non-removable product, primarily due to concern over being “stuck” with a product causing undesirable side-effects.

This study’s results are consistent with the finding that young women are willing to tolerate some side effects of a prevention product if they are counselled about them ahead of time, and develop a plan to address them [54]. With regards to oral PrEP, studies have found that gastrointestinal side effects only last for a brief time and with consumer-centered counseling prior to starting the product, clients are able to tolerate and manage the side-effects [55–57]. This DCE has reaffirmed the need to clarify and improve messaging around potential side effects with users up-front.

These DCE findings add to the increasing documented need to integrate FP and HIV prevention service delivery for AGYW in South Africa [58], and further research should be conducted into HIV-FP biomedical integration products to better examine family planning needs of HIV at-risk women and girls. Women in the survey stated a preference for a dual protection product for the prevention of HIV and unintended pregnancy over single independent products. This finding has been also discussed in previous surveys [59]. A recent study also demonstrated young women and men having sex with men also stated preference for PrEP injectable products [60]. However, we did not explore trade-offs (e.g. effectiveness, duration of protection, removability, etc.) between dual- and mono-protection options, and further research is needed to facilitate the next generation of multipurpose prevention technologies (MPT) products to address both HIV prevention and contraception in one product for target populations in Sub-Saharan Africa.

The study has contributed to the evidence that HIV at-risk women in South Africa desire a choice of highly-effective, discreet, convenient, and long-lasting biomedical HIV prevention products. Respondents in this study expressed a preference for a PrEP implant providing a year of protection over PrEP injectable products that are effective for 3 months. Globally, the findings demonstrate that a PrEP implant could resolve certain barriers of existing biomedical HIV prevention product options, potentially offering an avenue to improving AGYW’s uptake and adherence to a biomedical HIV prevention product and ultimately reducing HIV incidence in this high-risk group.

### Limitations

This study has several important limitations. First, PrEP implants remain in research and development and, for HIV prevention, they represented a new concept to all respondents. While the product was described using visual aids and a standard script, it is possible not all respondents understood the product or its characteristics in the same way. Additionally, though oral PrEP and the contraceptive implant are available in South Africa, awareness of—and more importantly—direct experience with these products were relatively low, especially among AG. As with all discrete choice experiments and other stated preference methods, there is a potential that the preferences expressed during the choice tasks may not reflect actual preferences or future observed behavior.

## Conclusion

The survey and DCE results demonstrate the importance of understanding at-risk end-user perspectives in designing, developing and scaling biomedical HIV prevention products. Providing AGYW with their preferred options and a range of product choices to control their HIV risk is a crucial step in the pathways to reduce HIV incidence in this priority population. All study groups expressed strong interest in adopting the theoretical PrEP implant, highlighting the need for continued investment in the design, development, testing, and scale-up of diverse biomedical HIV prevention products, especially long-acting ones, in high-incidence settings.

## Supplementary Information

Below is the link to the electronic supplementary material.

Supplementary file1 (DOCX 12 kb)

## Data Availability

The individual data is not published due to ethical constraints, however data from the study is available from the corresponding author with sponsor approval on reasonable request.

## Code Availability

The custom code generated to analyze data from the study is available from the corresponding author with sponsor approval on reasonable request.
