# Supplementary material for: HIV Pre-exposure Prophylaxis Implant Stated Preferences and Priorities: Results of a Discrete Choice Experiment Among Women and Adolescent Girls in Gauteng Province, South Africa
Source: AIDS Behav. 2022 Mar 31;26(9):3099–109. doi: 10.1007/s10461-022-03658-w (PMC9371991; doi:10.1007/s10461-022-03658-w)
Supplement: Supplementary file 1 — Supplementary file1 (DOCX 12 kb) [file 10461_2022_3658_MOESM1_ESM.docx]

**Supplementary Material 1:**

Sampling Approach Description

The study utilized a multi-stage sampling approach to respondent recruitment. The study areas in Gauteng were selected because they have had relevant interventions on PrEP implemented. Target audience in these areas therefore most likely familiar with PrEP which is relevant to this study. These two study areas were divided into rural and urban. AG and YW were sampled through randomized household sampling. Based on the list of households in each neighborhood area, obtained from national census, households were randomized. The first household was selected using simple random sampling through a random number generator. Subsequent households were based on the sampling interval. One eligible respondent was interviewed from each household. The Kish grid was used in cases where more than one eligible respondent was found in a given household.

FSW were sampled through respondent driven sampling, with the seed identified through observation in areas where FSWs frequent, such as bars and shebeens (unlicensed locations selling alcohol).
